# Characterization of the complete chloroplast genome of medical plant *Curculigo orchioides* Gaertn. (Amaryllidaceae)

**DOI:** 10.1080/23802359.2020.1863875

**Published:** 2021-01-27

**Authors:** Guobin Deng, Ruli Zhang, Junwen Yang, Chongli Deng, Yuan Zhang

**Affiliations:** a Yunnan Academy of Biodiversity, Southwest Forestry University, Kunming, China; b College of Biodiversity Conservation, Southwest Forestry University, Kunming, China; c College of Forestry, Southwest Forestry University, Kunming, China

**Keywords:** Amaryllidaceae, complete chloroplast genome, Illumina sequencing, phylogeny, Curculigo

## Abstract

*Curculigo orchioides* Gaertn. distributed in subtropical regions of Asia including southern China and India. The plant is used as a traditional medicine in China for the treatment of menorrhagia, osteoporosis, and other gynecological problems. The complete chloroplast genome was reported in this study using the Illumina NovaSeq platform. The whole genome of this species was 157,472 bp in length, with a total GC content of 37.44%. The large single copy (LSC) was 86,507 bp, the small single copy (SSC) was 16,867 bp, and both of the two inverted repeats (IRs) were 27,049 bp, respectively. A total of 132 unique genes were identified, among which are 86 protein-coding genes, 38 tRNA genes and 8 rRNA genes. The phylogenetic analysis revealed that *C. orchioides* was highly clustered with *C. capitulata*. Our study will provide useful fundamental data for further phylogenetic and evolutionary studies of *C. orchioides*.

*Curculigo orchioides* Gaertn. is a perennial herb of Amaryllidaceae, which grows widely in subtropical regions of Asia, such as southern China and India. The plant is used in the clinics of traditional Chinese and Indian medicine for many diseases such as osteoporosis and gynecological problems (Bafna and Mishra 2005; Jiao et al. 2009; Wang et al. 2012). The extracts of this plant contain a wide variety of phenolic glycosides, lignans, alkaloids, flavones, saponins, and other types of compounds (Valls et al. 2006; Zhou et al. 2020). The study also showed that the ethanol extracts of *C. orchioides* have the effect of strengthening the sexual behavior in male rats (Chauhan et al. 2007), and also have potential estrogenic activity in ovariectomized female albino rats (Vijayanarayana et al. 2007).

In recent years, with the increasing demand for *C. orchioides*, people have been digging and harvesting wild resources more immoderately. Moreover, because the rhizome of *C. orchioides* grows very slowly, and it has been threatened by habitat alternations, the resources of *C. orchioides* are significantly decreasing. For the above reasons, the medical effect and artificial cultivation of this plant have been intriguing the interests of researchers (Chen et al. 2017; Zhang et al. 2017; Zhou et al. 2020), while the complete chloroplast genome has not been sequenced. Considering the chloroplast DNA-based studies can provide invaluable data for studying genetic history and phylogeny, and can also provide important information in designing conservation and utilization strategies for the species, in this study, *C. orchioides* were collected from Laohuchong village, Qiubei county of Yunnan province (24°2773′ N, 103°8983′ E, 2228 m above sea level). A voucher specimen (YAB 202,007) was deposited at Yunnan Academy of Biodiversity, Southwest Forestry University, Yunnan, China. Then we sequenced, assembled and annotated the accurate chloroplast genome with the next-generation sequencing method, and the results will provide more useful information for phylogenetic and evolutionary research of this species.

For this study, the total genomic DNA of *C. orchioides* was extracted from fresh leaves according to the modified CTAB methods (Doyle and Doyle 1987). A genomic shotgun library with an insertion size of 341 bp was constructed, the libraries were sequenced on Illumina NovaSeq platform at Personalbio Biotech (Shanghai, China). The chloroplast genome was assembled using GetOrganelle software version 1.7.1 (Jin et al. 2020), and the assembled chloroplast genome was annotated through the online program CPGAVAS 2 (Shi et al. 2019) with *C. capitulata* chloroplast genome (GenBank accession number: MT610372) as a reference, and assisted with manual correction. The raw sequencing reads used in this study have been deposited in SRA (accession number: SRR12793630) and the annotated chloroplast genome sequence has been deposited into the GenBank (accession number: MW079480).

The complete chloroplast genome of *C. orchioides* was 157,472 bp and composed of two IRs of 27,049 bp each, which divide a large single copy (LSC) region of 86,507 bp and a small single copy (SSC) region of 16,867 bp, the average GC content was 37.44%, with IR regions (42.53%) higher than that in LSC (35.44%) and SSC regions (31.37%). The chloroplast genomes encoded 132 unique genes, including 86 protein-coding genes, 38 tRNA genes, and 8 rRNA genes. A total of 86 SSR markers ranging from mononucleotide to pentanucleotide repeat motif were identified in the chloroplast genome of *C. orchioides*. The intron-exon structure analysis indicated that 17 genes have introns, among which *atpF, ndhA, ndhB, petB, petD, rpl16, rpl2, rpoC1, rps16, trnA-UGC, trnG-UCC, trnI-GAU, trnK-UUU, trnL-UAA* and *trnV-UAC* have one intron, while *ycf3* and *clpP* have two introns.

To determine the phylogenetic relationship of *C. orchioides*, based on complete chloroplast genomes of the other 18 species within the family Amaryllidaceae (Figure 1), chloroplast genomes were downloaded from NCBI. All chloroplast genomes were aligned using the program MAFFT v7.471 (Rozewicki et al. 2019), and phylogenetic tree (maximum likelihood) constructed by Iqtree software version 1.6.12 (Minh et al. 2020) with 1000 bootstrap replicates, best-fitted model has been confirmed is TVM + F+R2 by ModelTest-NG (Darriba et al. 2020). The phylogenetic analysis revealed that *C. orchioides* closely clustered with *C. capitulata*. The study will provide essential data for future research on the phylogenetic and evolutionary relationship of *C. orchioides* and the family Amaryllidaceae.

**Figure 1.**
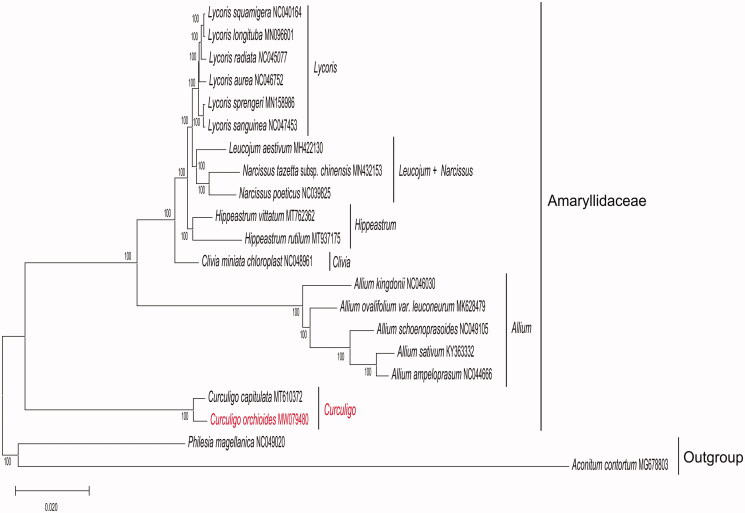
Maximum likelihood (ML) phylogenetic tree based on complete chloroplast genomes of 19 representative species of Amaryllidaceae and 2 outgroups. Numbers at nodes represent bootstrap values. The genome sequence in this study is highlighted with red text.

## Data Availability

The genome sequence data that support the findings of this study are openly available in GenBank of NCBI at [https://www.ncbi.nlm.nih.gov] (https://www.ncbi.nlm.nih.gov/) under the accession no. MW079480. The associated BioProject, SRA and Bio-Sample numbers are PRJN667995, SRR12793630, and SAMN16393142 respectively.
